# Congestion and systemic inflammation in acute heart failure: correlations and prognostic role

**DOI:** 10.3389/fcvm.2025.1695500

**Published:** 2025-11-17

**Authors:** Pietro Scicchitano, Daniele De Feo, Massimo Iacoviello, Stefano Albani, Gabriella Ricci, Anna Livrieri, Cosimo Campanella, Pasquale Caldarola, Marco Matteo Ciccone, Francesco Massari

**Affiliations:** 1 Cardiology Section, Hospital “F. Perinei”, Bari, Italy; 2 Cardiology Section, Hospital “S. Paolo”, Bari, Italy; 3 Cardiology Section, University of Foggia, Foggia, Italy; 4 Division of Cardiology, U. Parini Hospital, Aosta, Italy; 5 Department of Cardiology, Cardiovascular Institute Paris Sud, Massy, France; 6 Cardiology Section, University of Bari “A. Moro”, Bari, Italy

**Keywords:** acute heart failure, congestion, inflammation, high-sensitivity C-reactive protein, neutrophil–lymphocyte ratio

## Abstract

**Background:**

The reciprocal correlation between systemic inflammation and heart failure (HF) is a hotline research topic although the exact role in risk stratification and prognosis within the acute setting is a matter of debate. This study aimed to evaluate the correlation among two inflammatory biomarkers—namely, high-sensitivity C-reactive protein (hs-CRP) and neutrophil–lymphocyte ratio (NLR), congestion status, and prognosis in patients with acute HF (AHF).

**Methods:**

We consecutively enrolled 314 AHF patients. Congestion biomarkers [brain natriuretic peptide, estimated plasma volume status (ePVS), hydration index (HI), blood urea nitrogen to creatinine ratio (BUN/Cr)] were evaluated to assess hemodynamic intravascular, peripheral, and venous congestion. We also measured hs-CRP and NLR as markers of inflammation. The endpoint was all-cause mortality at 90 days.

**Results:**

hs-CRP concentrations at admission [12.1 mg/L, 95% confidence interval (CI) 10–15] were associated with NLR values (4.8, 95% CI: 4.3–5.3) (*R*^2^ = 0.11; *p* < 0.0001). They both positively correlated with congestion indexes (log hs-CRP, ePVS *r* = 0.2, HI *r* = 0.24, *p* < 0.0001 for both; log NLR, ePVS *r* = 0.20, HI *r* = 0.29, BUN/Cr 0.14, log brain natriuretic peptide (BNP) *r* = 0.16, *p* < 0.01 for all). After 90 days, the cumulative mortality rate was 26%. Inflammatory biomarkers (hs-CRP and NLR cutoffs were >60 pg/mL and >7.5, respectively) were predictors of death. By using all these parameters, we performed an index of inflammation (from 0 to 2) for each patient. Congestion and inflammation indexes were independent predictors of mortality (hazard ratios 1.4 and 2.3, respectively; C-index 0.72).

**Conclusion:**

Systemic inflammation seemed directly associated with congestion burden in patients with AHF. Both of them have different impacts on the prediction of adverse outcomes in these patients. Further studies are needed to address unmet needs.

## Introduction

Acute heart failure (AHF) remains a major cause of hospitalization and mortality worldwide, representing a complex clinical syndrome characterized by the rapid onset or worsening of symptoms and signs of heart failure (HF), often requiring urgent therapeutic intervention (1, 2). Despite advances in the management of chronic HF, outcomes following an episode of AHF remain poor, with high rates of in-hospital complications, early readmission, and death (1, 2). This underscores the urgent need for improved strategies for early risk stratification and individualized prognostication (3).

Two pivotal pathophysiological processes—congestion and inflammation—have emerged as central determinants of clinical trajectory and outcome in AHF (4–6). Congestion, defined as the accumulation of fluid due to elevated cardiac filling pressures, is the hallmark of AHF and the primary reason for hospitalization (7, 8). However, evidence suggests that traditional clinical assessments—namely, signs and symptoms of congestion such as dyspnea on exertion, orthopnea, edema, resting jugular vein distension, chest x-ray, cardiomegaly, redistribution, interstitial edema, or pleural effusion—often underestimate the degree of hemodynamic and tissue congestion, leading to suboptimal decongestion and poor prognosis (8). Certainly, multiparametric approaches might improve the overall evaluation and risk stratification of patients with AHF (9, 10).

Parallel to congestion, inflammation has gained increasing attention as a key player in AHF (6). Inflammatory activation, whether triggered by myocardial injury, neurohormonal dysregulation, or peripheral organ dysfunction, contributes to endothelial damage, myocardial fibrosis, and multiorgan failure (6). Emerging data suggest a bidirectional relationship, whereby congestion may exacerbate systemic inflammation through gut ischemia, bacterial translocation, and neurohormonal activation, while inflammation may impair natriuresis and contribute to persistent fluid overload (11, 12). Data from the Studies of Left Ventricular Dysfunction (SOLD) trial (13) outlined the increase in plasma levels of tumor necrosis factor-alpha (TNF-α) and interleukin-6 (IL-6) in patients with HF, their concentrations rising in relation to the worsening of the HF condition. Studies revealed the negative impact of cytokines on left ventricular remodeling, promotion of cardiac contractility impairment, and negative influence on myocardial β-adrenergic receptors (14–16). The infusion of recombinant human (rh) tumor necrosis factor-alpha (TNF-alpha) in dogs effectively impaired their LV systolic and diastolic function (14). Similarly, hamster papillary muscles were blocked in contractility when TNF-α, IL-6, and IL-2 were administered (15).

High-sensitivity C-reactive protein (hs-CRP) (6) and neutrophil–lymphocyte ratio (NLR) (17) have been raised as representative inflammatory biomarkers in AHF, mainly related to prognosis, as they both correlate to the severity and progression of disease.

To date, the literature lacks definite data about the connection among AHF, congestion, and hs-CRP/NLR and the impact of these variables and biomarkers on the prognosis of AHF.

The present study aims to investigate the combined role of congestion and inflammation in the risk stratification and short- to mid-term prognosis of patients hospitalized for AHF. By integrating clinical, biochemical, and imaging markers of both processes, we seek to identify profiles associated with increased risk and to explore potential mechanistic links that could inform tailored therapeutic strategies.

## Material and methods

### Study patients

This was a retrospective study. We reviewed clinical data of all consecutive patients who were hospitalized with AHF between January 2020 and September 2024. Inclusion criteria were hospitalization for AHF and age >18 years. Exclusion criteria included acute myocarditis, acute pulmonary embolism, acute coronary syndrome, recent cardiac surgery intervention, coronavirus disease or other inflammatory/infective conditions, history of active neoplasm, previous heart transplantation, hemodialysis, and the use of corticosteroids/antibiotics.

Patients' baseline anthropometric characteristics, comorbidities, blood biochemical data, and pharmacological background were collected. AHF was defined as acute decompensation of chronic heart failure or *de novo* acute heart failure (*de novo* AHF). We assessed left ventricular ejection fraction (LVEF) by transthoracic echocardiography and defined it as “preserved” when LVEF was (≥50%). At admission, we assessed hydration index (HI, %) as derived by bioimpedance vector analysis (BIVA) (Bodygram 1.4, Akern RJL Systems, Florence, Italy) and estimated plasma volume status [ePVS, by means of Strauss–Duarte's formula: (100 − hematocrit %) / hemoglobin (g/dL) (9)]. We measured the plasma concentrations of hs-CRP (Beckman Coulter AU680), brain natriuretic peptide (BNP) (Architect, Abbott Park, IL, USA), and blood urea nitrogen–creatinine ratio (BUN/Cr ratio). NLR was calculated by dividing the neutrophil count by the lymphocyte count. As previously reported, according to the cutoffs of BNP, ePVS, HI, and BUN/Cr (>441 pg/mL, >5.3 dL/g, >73.8%, and >25, respectively), we generated the HYDRA score with a range from 0 to 4 (9). The estimated glomerular filtration rate (eGFR) level was calculated using the modified Modification of Diet in Renal Disease (MDRD) equation.

The primary endpoint was all-cause mortality at 90 days, which was assessed from available medical records or National Death Records. The study complied with the Declaration of Helsinki and was approved by the local Institutional Review Board. Written informed consent was obtained from each patient at inclusion (protocol no. 0081801/CE, 29/10/2015; study number: 4816).

### Statistical analysis

Normally distributed variables were expressed as mean ± standard deviation, and non-normally distributed continuous variables were expressed as median (95% CI). Variables non-normally distributed (Shapiro–Wilk test) were log-transformed for use in analyses. Univariable Pearson correlation was used to assess the significance and magnitude of relationships among biomarkers, i.e., congestion and inflammation. Collinearity for each variable significant in the univariate analyses was analyzed by calculating variance inflation factors (VIF). The VIF values lower than 4 were considered acceptable for excluding multicollinearity. Differences between the groups were evaluated by a paired Student’s *t*-test or a one-way ANOVA. Receiver-operating characteristic (ROC) curve analysis was performed to calculate the area under the curve (AUC) values and Youden index to assess the optimal cutoff values for mortality. Kaplan–Meier survival curve with log-rank significance test was also performed. Uni- and multivariate analyses were performed by Cox for proportional hazards and used to analyze the factors associated with all-cause mortality, calculate hazard ratios (HR), and 95 CI. *p*-values below 0.05 were defined as statistically significant using STATA software, version 12 (StataCorp, College Station, TX, USA).

## Results

The baseline characteristics of the study population at admission are shown in Table 1.

**Table 1:** Characteristics of the study population.

Characteristics	Data
Age, years	80 ± 11
Male, %	52
Body mass index, kg/m^2^	29 ± 15
*De novo* AHF %	38
Heart rate, bpm	87 ± 24
Systolic blood pressure, mmHg	126 ± 2
NYHA III/IV, %	61/39
Medical history, %	
Coronary artery disease	30
Diabetes	30
Atrial fibrillation	43
COPD	41
PM	12
ICD	17
Laboratory values	
LVEF, %	40 ± 13
Preserved EF	38
Hemoglobin, g/dL	12 ± 2
White blood cell count (×1,000/uL)	7.9 ± 3.7
BUN, mg/dL	37 ± 23
Creatinine, mg/dL	1.5 ± 0.9
eGFR, mL/min/1.73 m^2^	48 ± 23
Sodium, mmol/L	139 ± 5
Potassium, mmol/L	4.1 ± 0.5
Biomarkers of inflammation	
hs-CRP, mg/L	12.1 (10.5–14.7)
NLR	4.8 (4.3–5.2)
Biomarkers of congestion	
BNP, pg/mL	1,120 (951–1,211)
ePVS, dL/g	5.1 ± 1.6
Hydration index, %	80 ± 6.5
BUN/creatinine	24 ± 9
Therapies (%)	
IV furosemide	100
Beta-blockers	66
ACE inhibitors/ARB	40
ARNI	5
MRA	77
SGLT2i	8
Digitalis	13
IV inotropes	26
NIV	32
Length of hospital stay, days	6.9 (6.4–7.4)

ACE, angiotensin-converting enzyme; AHF, acute heart failure; ARB, angiotensin receptor blocker; ARNI, angiotensin receptor II blocker neprilysin inhibitor; BNP, brain natriuretic peptide; BUN, blood urea nitrogen; COPD, chronic obstructive pulmonary disease; eGFR, estimated glomerular filtration rate; NLR, neutrophil–lymphocyte ratio; hs-CRP, high-sensitivity C-reactive protein; ICD, implanted cardioverter/defibrillator; IV, intravenous; LVEF, left ventricular ejection fraction; MRA, mineralocorticoid receptor antagonist; NIV, non-invasive ventilation; PM, pacemaker; SGLT2i, sodium–glucose cotransporter 2 protein inhibitor.

**Figure 1:**
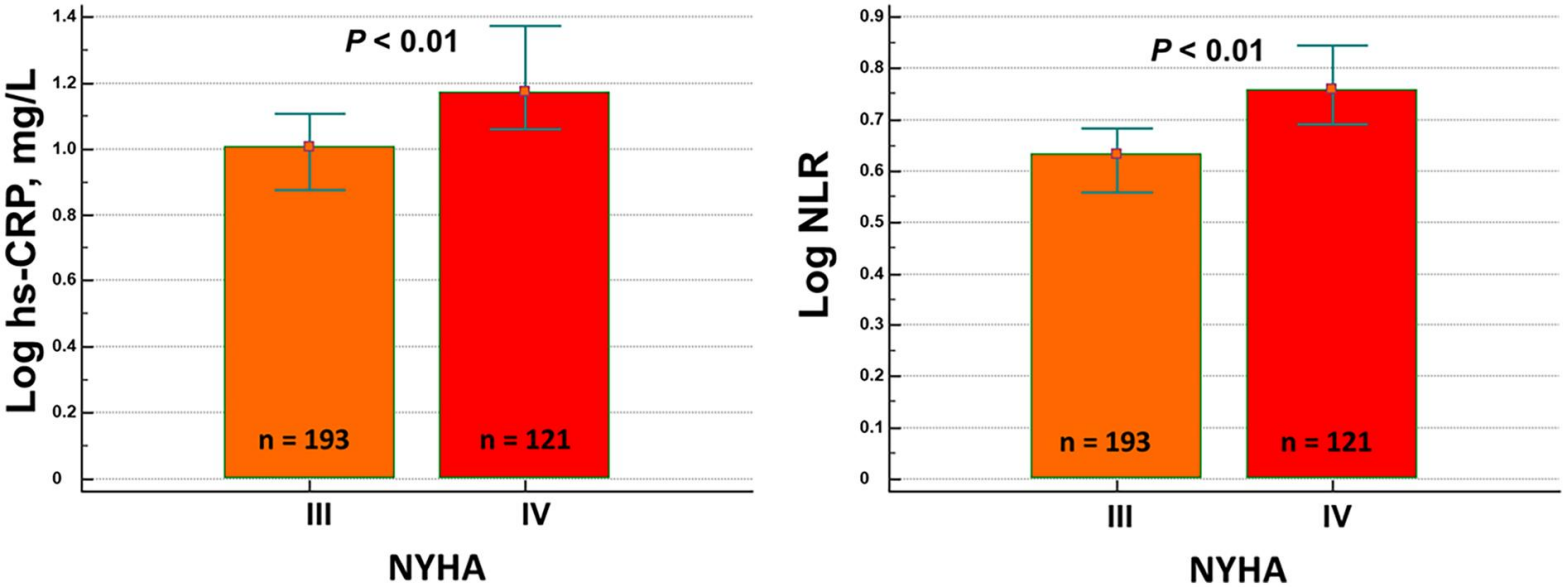
Correlation between high-sensitivity C-reactive protein (hs-CRP) and neutrophil–lymphocyte ratio (NLR).

**Figure 2:**
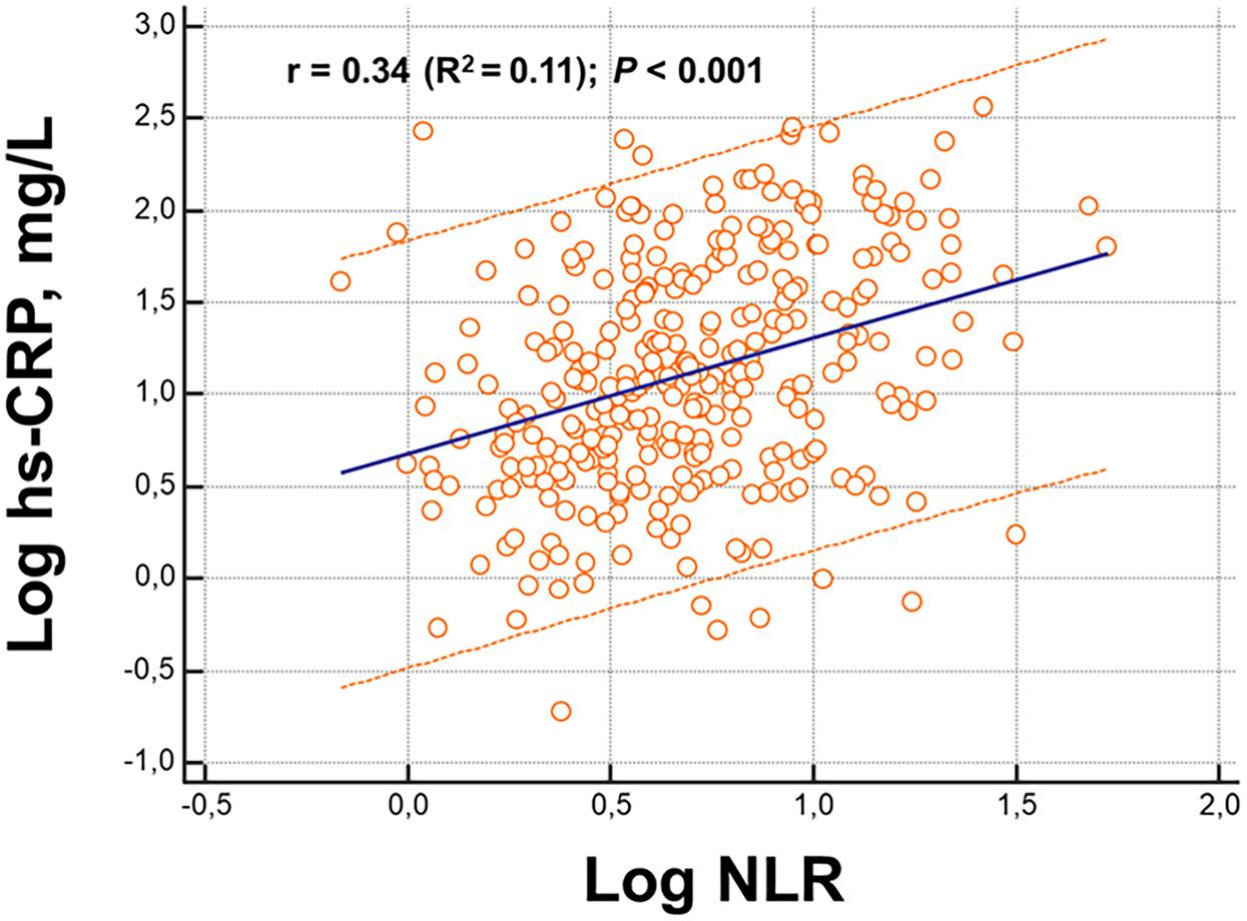
Relationship between high-sensitivity C-reactive protein (hs-CRP) and neutrophil–lymphocyte ratio (NLR) and NYHA functional class.

NLR and hs-CRP were related to each other, although the *R*^2^ was low (=0.11 and VIF = 1.01) (Figure 1). The increase in their plasma concentration was positively associated with the length of hospital stay (*r* = 0.24; *p* < 0.001; for both) and worsening New York Heart Association (NYHA) functional class (Figure 2).

We tried to evaluate the relationship between inflammatory and congestion biomarkers.

**Figure 3:**
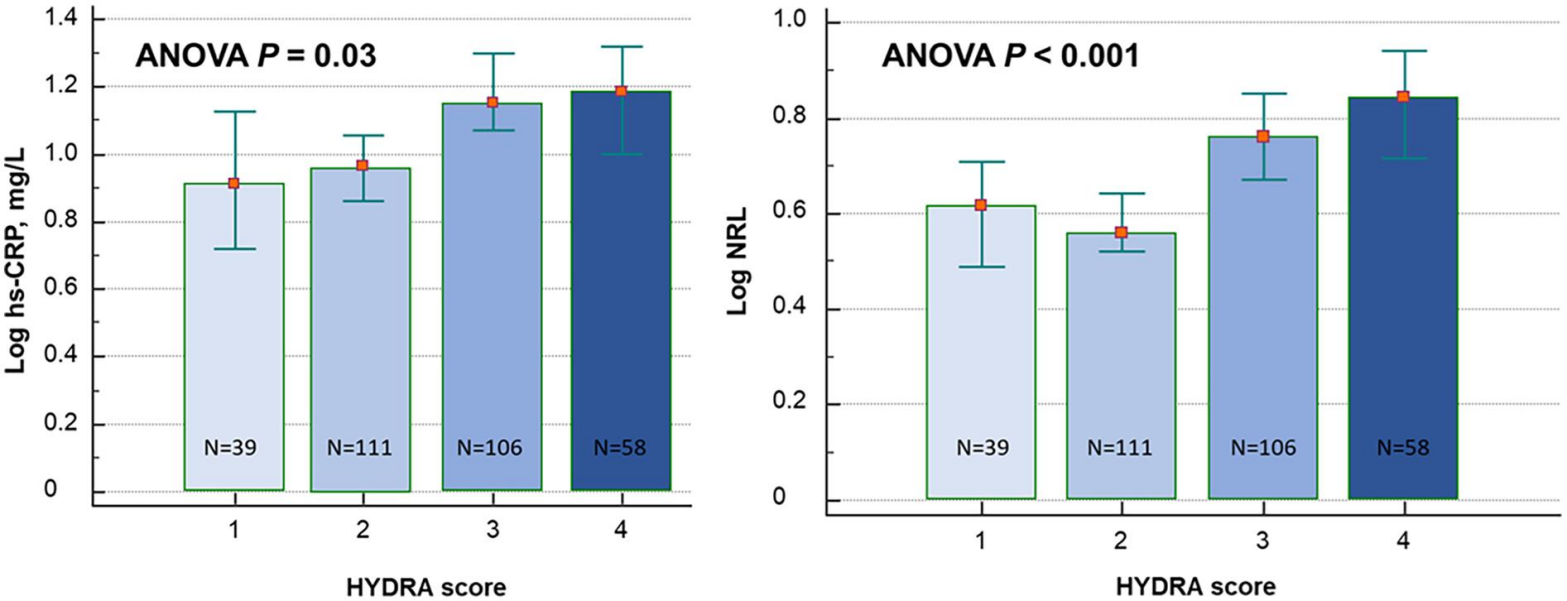
Relationship between high-sensitivity C-reactive protein (hs-CRP) and neutrophil–lymphocyte ratio (NLR) and HYDRA score.

Positive correlations were among logNLR and all four biomarkers of congestion (viz., ePVS, HI, BUN/Cr ratio, and log BNP), while log hs-CRP was related to HI and ePVS (Table 2). Therefore, we categorized the congestion status of the patients via the HYDRA score (9). Figure 3 represented the relationship between HYDRA score and inflammatory biomarkers: the higher the HYDRA score, thus the burden of the overall congestion of the patient, the higher the log values of NLR and hs-CRP.

**Table 2:** Pearson correlation between biomarkers of inflammation and congestion.

Biomarkers	Log hs-CRP, pg/dL	Log NLR
ePVS, dL/g	*r* = 0.20	*r* = 0.20
	*p* < 0.0001	*p* = 0.0004
HI, %	*r* = 0.24	*r* = 0.29
	*p* < 0.0001	*p* < 0.0001
BUN/Cr	*r* = 0.03	*r* = 0.14
	*p* = 0.5	*p* = 0.01
Log BNP	*r* *=* 0.05	*r* = 0.16
	*p* *=* 0.4	*p* = 0.01

BNP, brain natriuretic peptide; BUN/Cr, blood urea nitrogen–creatinine ratio; ePVS, estimated plasma volume status; HI, hydration index; hs-CRP, high-sensitivity C-reactive protein; NLR, neutrophil–lymphocyte ratio.

At 90-day follow-up, 81 patients (26%) died after an average of 34 days (95% CI: 27–42). The median NLR value was lower in survivors (4.4; 95% CI: 3.9–4.8) than in patients who died (7.1; 95% CI: 5.4–8.4; *p* < 0.001). The median hs-CRP plasma concentrations were also significantly lower in survivors (10.6; 95% CI: 5.4–8.4) than in patients who died (22.7; 95% CI, 13.9–42.1; *p* < 0.001).

Both inflammatory biomarkers were predictors of mortality at 90 days (AUC 0.65 and *p* < 0.001 for both), and optimal cutoff values were observed to be >7.5 and >60 pg/mL, respectively. Patients were then stratified into three groups based on NLR and hs-CRP optimal cutoff values at admission. We noticed that 204 of them showed no inflammatory biomarkers (score 0), 80 out of 214 had only one (score 1), and 30 of them presented with two increased parameters (score 2) (inflammatory index).

**Figure 4:**
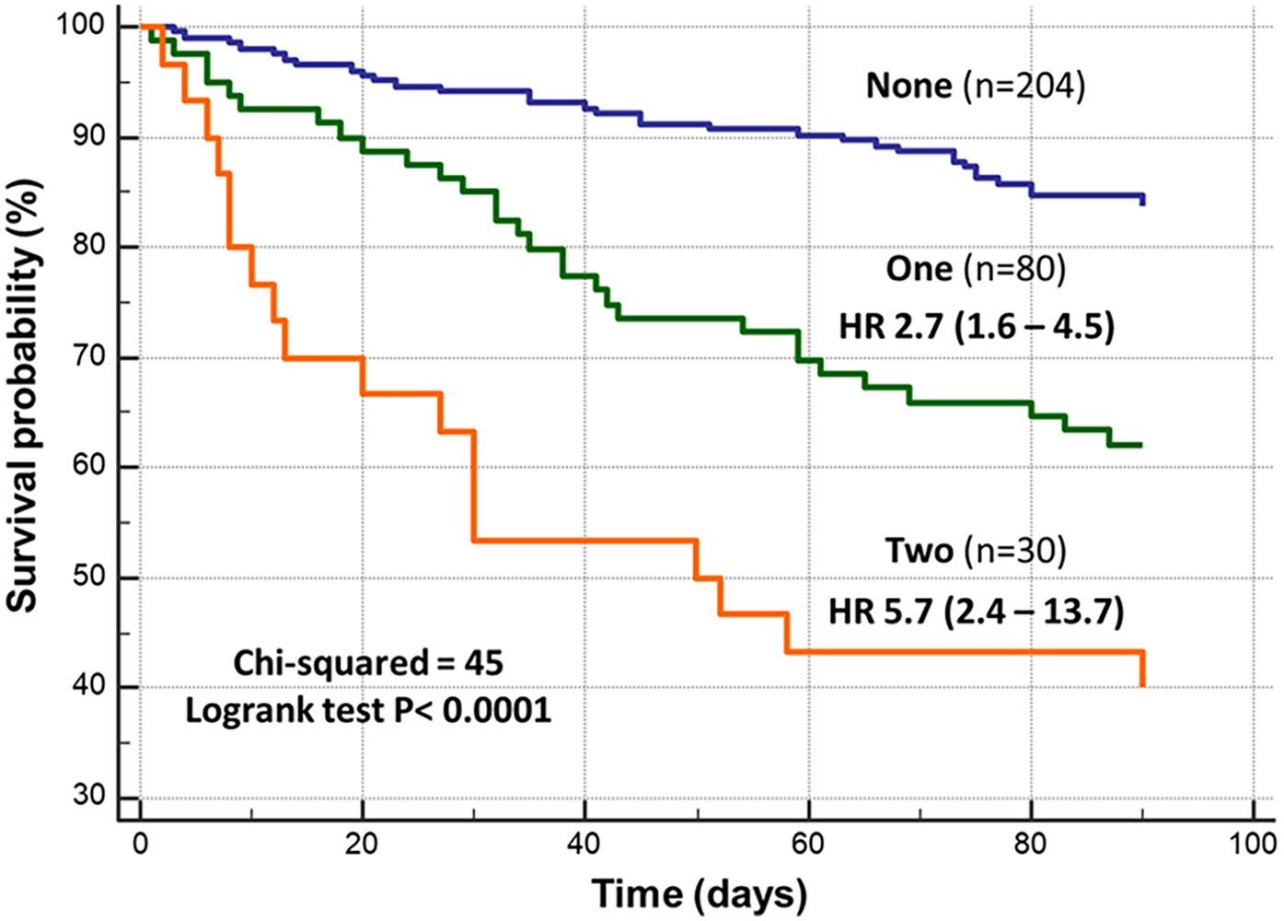
Kaplan–Meier curves related to the presence of none (*n* = 204), one (*n* = 80), or two (*n* = 30) inflammatory biomarkers above the optimal cutoff. HR, hazard ratio (95% CI) vs. group with no biomarker of inflammation.

In the univariate Cox analysis, HYDRA score, NLR, and hs-CRP were all predictors of death, both individually or collectively considered (Table 3). Figure 4 shows the Kaplan–Meier survival curves. The curves were proportionally related to the presence of none, one, or two biomarkers of inflammation. The observed mortality rates were 13%, 37%, and 60%, respectively. The risk for death was nearly threefold higher in patients with at least one elevated biomarker and almost sixfold higher in those patients presenting with higher values of the two inflammatory biomarkers.

**Table 3:** Predictive value of biomarkers of inflammation and congestion.

Variables	Univariate analysis		Multivariate analysis		C-index
	HR (95% CI)	*p*	HR (95% CI)	*p*	
Model 1					
HYDRA score (from 1 to 4)	1.65 (1.22–2.32)	0.003	1.45 (1.05–2.01)	0.02	0.70 (0.64–0.75)
hs-CRP, mg/L ×10	1.06 (1.03–1.12)	0.0001	1.05 (1.006–1.01)	0.004	
NLR	1.09 (1.05–1.11)	<0.0001	1.06 (1.03–1.09)	<0.0001	
Model 2					
HYDRA score (from 1 to 4)	1.65 (1.22–2.32)	0.003	1.47 (1.07–2.01)	0.02	0.72 (0.66–0.77)
Inflammatory index (from 0 to 2)	2.42 (1.83–1.3.2)	<0.0001	2.30 (1.73–3.04)	<0.0001	

CI, confidential interval; hs-CRP, high-sensitivity C-reactive protein; HR, hazard ratio; NLR, neutrophil–lymphocyte ratio.

In the multivariate Cox analyses, congestion and inflammation biomarkers remained independent predictors of mortality (Table 3).

## Discussion

In this retrospective study, we demonstrated that inflammatory biomarkers—NLR and hs-CRP—are significantly associated with congestion burden and are independent predictors of short-term mortality in patients hospitalized for AHF. These findings support the hypothesis that congestion and inflammation are interrelated pathophysiological processes in AHF and their combined evaluation can enhance risk stratification and prognostication.

Our data showed that both NLR and hs-CRP increased proportionally with the severity of congestion, as estimated by the HYDRA score, which integrates four validated markers of volume overload (BNP, BUN/Cr ratio, estimated plasma volume status, and hydration index) (9). The positive correlations between inflammatory and congestion biomarkers suggest a potential pathophysiological interplay, where congestion may serve as a trigger or amplifier of systemic inflammation. Indeed, hs-CRP could not be used interchangeably with NLR when dealing with congestion status: hs-CRP correlated only with two of the congestive biomarkers, while NLR was related to all of them. Importantly, elevated NLR and hs-CRP levels at admission were associated with longer hospital stays, worsening functional class, and increased mortality at 90 days. Kaplan–Meier analyses demonstrated a stepwise increase in mortality with rising inflammatory burden, with death rates ranging from 13% in patients with no elevated biomarkers to 60% in those with both NLR and hs-CRP above the identified cutoff values.

Although the influence of systemic inflammation and congestion—taken separately—in HF has been widely developed in the literature (6, 18, 19), the combination of both of them in risk stratification and prognostic evaluation of patients with AHF is still a matter of debate.

We already demonstrated the impact of combining congestion biomarkers in both chronic and acute HF (9): the combination of BNP, ePVS, BIVA, and BUN/Cr at admission—unified into the HYDRA score—might effectively predict death in these patients independently from confounding factors (HR, 2. 1; HR, 2.2; HR, 2.1; and HR, 1.7; C-index 0.756). Specifically, when bioimpedance analysis—and phase angle in particular—was associated with BNP, BUN, and partial pressure in oxygen, it effectively explained 60% of death in AHF (20). Congestion biomarkers are a large expression of subtle damage to kidney function, which might impact the overall risk of death of these patients (21, 22). A large cohort of Spanish patients—approximately 18,120—revealed an increased risk of death by 109%, 123%, and 156% in patients with 1–2, 3–5, and 6–7 symptoms/signs of congestion, respectively (23).

Studies (24–26) tried to evaluate the prognostic impact of hs-CRP in the acute setting of HF. Santas et al. (27) demonstrated the role of hs-CRP in predicting increased risk of long-term death and total HF readmissions in patients with AHF. The ATTEND study (26) found a 44% increase in short-term cardiac and non-cardiac mortalities in patients with AHF and markedly elevated CRP levels (>11.8 mg/L). Similar results were obtained when considering NLR (28). Patients with higher NLR values demonstrated a 2.2-fold increase in mortality and a 3.5-fold increase in 30-day readmission rate in patients who were admitted for acute decompensated heart failure (ADHF) (29). Data from the BIOlogy Study to Tailored Treatment in Chronic Heart Failure (BIOSTAT-CHF) study suggested that higher NLR values significantly predicted all-cause mortality or HF hospitalization in patients with worsening or new-onset HF, independently of the type of HF (30). A recent meta-analysis (17) confirmed these findings: by collecting data from 15,995 patients with AHF, the authors found a 54% increase in in-hospital and 61% increase in long-term all-cause mortality, respectively, in patients with the highest values of NLR. Moreover, the chronic setting revealed the predictive value of NLR within HF prognosis: lower values are in those with reduced and mildly reduced ejection fraction (EF) as compared to controls, the AUC of NLR being higher than 0.7 (31).

The two inflammatory biomarkers are poorly related to each other in AHF. Our study demonstrated a correlation of *r* = 0.34, which was similar to the finding from Yang et al. (*r* = 0.35) (32). Nevertheless, taken together, they became a reliable tool for better stratifying the risk of these patients. Our inflammatory index and the C-NLR score from Yang et al. (32) effectively revealed the independent role of both of these biomarkers in predicting mortality in patients with AHF.

The inflammatory index we proposed—based on the presence of none, one, or two elevated inflammatory biomarkers—further improved prognostic discrimination, identifying a subgroup of patients at very high risk for early mortality. When integrated with congestion burden, as assessed by the HYDRA score (9), the prognostic value of inflammation became even more evident. Multivariate Cox regression analysis confirmed that both congestion and inflammation independently contribute to mortality risk in AHF, underscoring the need for a multiparametric approach in the clinical evaluation of these patients.

This is the main novelty of our paper: integration between congestion and inflammation for the prediction of adverse events in AHF. Pellicori et al. (33) recently reported that increased plasma concentration of hs-CRP in chronic heart failure was associated with higher values of BNP as well as signs of clinical congestion (peripheral edema, lung crackles, and increased jugular venous pressure). Indeed, the relationship between BNP levels and hs-CRP and/or NLR in AHF is not clarified. Contrasting results were for hs-CRP (25–27, 34), while some studies (29, 30) reported a direct correlation between congestion status and NLR. Indeed, we did not perform an integrated score including both inflammatory and congestion biomarkers. A combined score would certainly improve the prognostic evaluation of patients with HF, although further studies with a wider sample size are needed.

Our findings provide a compelling rationale for routinely incorporating both congestion and inflammation biomarkers into clinical decision-making processes. While congestion has long been the primary therapeutic target in AHF, our results highlight that inflammation may represent a complementary and under-recognized determinant of adverse outcomes. This dual-pathway model opens new perspectives for tailored management strategies. For instance, future studies should evaluate whether patients with high inflammatory burden might benefit from specific anti-inflammatory interventions or closer post-discharge monitoring.

## Limitation

Limitations must be acknowledged. First, the retrospective design of our study limits causal inference. Second, although our exclusion criteria aimed to minimize confounding from other inflammatory conditions, subclinical or undetected sources of inflammation cannot be completely ruled out. Indeed, we definitely excluded the presence of overt inflammatory and infective diseases, but there was no possibility of excluding further subclinical causes of infection. We are completing a dedicated dataset of a prospective, observational study dealing with the comprehensive evaluation of congestion status in patients with HF. This would try to enforce the conclusions of this study.

Third, we did not assess changes in biomarker levels during hospitalization or post-discharge, which could provide further insights into disease trajectory and treatment response. Finally, the cohort is elderly and from a single geographic region, which might generate difficulties in generalizing conclusions to younger populations and different healthcare settings. Therefore, data and results should be validated in larger, multicenter, and more diverse cohorts.

## Conclusions

In conclusion, our study reinforces the central role of congestion and inflammation in the pathophysiology and prognosis of AHF. The combined assessment of NLR, hs-CRP, and HYDRA score provides a robust and clinically relevant strategy for early risk stratification. Further prospective studies are warranted to validate our findings and to explore whether targeted therapeutic strategies addressing both congestion and inflammation can improve clinical outcomes in this high-risk population.

## Data Availability

The raw data supporting the conclusions of this article is available from the corresponding author upon request.
